# Flutolanil [*N*-(3-isopropoxyphen­yl)-2-(trifluoro­meth­yl)benzamide]

**DOI:** 10.1107/S1600536810034422

**Published:** 2010-09-04

**Authors:** Hyunjee Kim, Hojin Yang, Jae Sang Kim, Suk-Hee Moon, Tae Ho Kim

**Affiliations:** a Department of Chemistry and Research Institute of Natural Sciences, Gyeongsang National University, Jinju 660-701, Republic of Korea; b Subdivision of Food Science, Kyungnam College of Information and Technology, Busan 616-701, Republic of Korea

## Abstract

The title compound, C_17_H_16_F_3_NO_2_, crystallizes with two independent mol­ecules in the asymmetric unit. The dihedral angles between the isopropoxyphenyl and trifluoro­methyl­phenyl rings are 85.78 (5) and 63.15 (6)° in the two mol­ecules. In the crystal structure, inter­molecular N—H⋯O and C—H⋯π inter­actions are observed.

## Related literature

For information on the toxicity and fungicidal properties of the title compound, see: Uchida *et al.* (1983 ▶). For related structures, see: Balasubramanyam *et al.* (2003 ▶); Saeed *et al.* (2008 ▶).
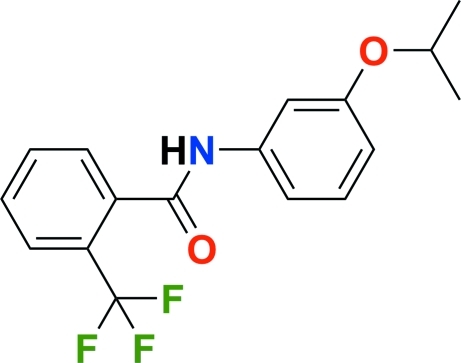


## Experimental

### 

#### Crystal data

C_17_H_16_F_3_NO_2_
*M* *_r_* = 323.31
Triclinic, 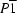

*a* = 9.3264 (3) Å
*b* = 11.9326 (3) Å
*c* = 15.2208 (3) Å
α = 71.125 (2)°
β = 87.493 (2)°
γ = 79.495 (2)°
*V* = 1575.73 (7) Å^3^
*Z* = 4
Mo *K*α radiation
μ = 0.11 mm^−1^
*T* = 173 K
0.29 × 0.25 × 0.20 mm

#### Data collection

Bruker SMART APEXII CCD diffractometer
Absorption correction: multi-scan (*SADABS*; Sheldrick, 1996 ▶) *T*_min_ = 0.968, *T*_max_ = 0.978
14333 measured reflections
6778 independent reflections
5306 reflections with *I* > 2σ(*I*)
*R*_int_ = 0.025

#### Refinement

*R*[*F* ^2^ > 2σ(*F* ^2^)] = 0.042
*wR*(*F* ^2^) = 0.111
*S* = 1.08
6778 reflections
419 parameters
H-atom parameters constrained
Δρ_max_ = 0.21 e Å^−3^
Δρ_min_ = −0.25 e Å^−3^

### 

Data collection: *APEX2* (Bruker, 2006 ▶); cell refinement: *SAINT* (Bruker, 2006 ▶); data reduction: *SAINT*; program(s) used to solve structure: *SHELXTL* (Sheldrick, 2008 ▶); program(s) used to refine structure: *SHELXTL*; molecular graphics: *SHELXTL* and *DIAMOND* (Brandenburg, 1998 ▶); software used to prepare material for publication: *SHELXTL*.

## Supplementary Material

Crystal structure: contains datablocks global, I. DOI: 10.1107/S1600536810034422/lx2169sup1.cif

Structure factors: contains datablocks I. DOI: 10.1107/S1600536810034422/lx2169Isup2.hkl

Additional supplementary materials:  crystallographic information; 3D view; checkCIF report

## Figures and Tables

**Table 1:** Hydrogen-bond geometry (Å, °) *Cg* is the centroid of the C26–C31 isopropoxyphenyl ring.

*D*—H⋯*A*	*D*—H	H⋯*A*	*D*⋯*A*	*D*—H⋯*A*
N1—H1*N*⋯O3	0.88	2.08	2.8875 (15)	153
N2—H2*N*⋯O1^i^	0.88	1.95	2.7984 (16)	161
C15—H15⋯*Cg*	1.00	2.69	3.53	142

Symmetry code: (i) 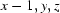.

**Fig. 1.**
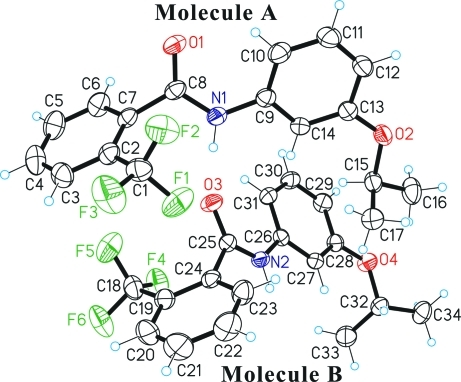
The molecular structure of the title compound with the atom numbering scheme. Displacement ellipsoids are drawn at the 50% probability level. H atoms are presented as a small spheres of arbitrary radius.

**Fig. 2.**
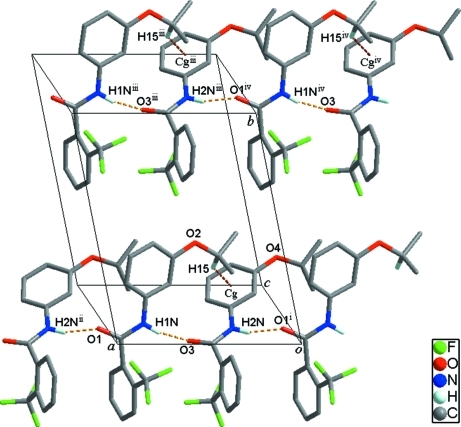
N—H···O and C—H···π interactions (dotted lines) in the crystal structure of the title compound. Cg denotes the ring centroid. [Symmetry codes: (i) x - 1, y, z; (ii) x + 1, y, z; (iii) x, y + 1, z; (iv) x - 1, y + 1, z.]

## supplementary crystallographic information

### Comment

Flutolanil [systematic name: *N*-(3-isopropoxyphenyl)-2-(trifluoromethyl)benzamide] is well known as a fungicide which shows protective and curative effects against many crop diseases (Uchida *et al.*, 1983). However it's crystal structure has not been reported yet. Here we report the crystal structure of the title compound, which has unique molecules in the asymmetric unit (further marked as A & B) (Fig. 1).

The dihedral angles between the isopropoxyphenyl and the trifluoromethylphenyl rings are 85.78 (5) and 63.15 (6)° in the molecules A and B, respectively. The molecular packing (Fig. 2) is stabilized by intermolecular N—H···O hydrogen bonds between the amide groups from adjacent molecules (Table 1). The molecular packing (Fig. 2) is further stabilized by an intermolecular C—H···π interaction between a methine H atom of the isopropyl group and the isopropoxyphenyl ring of a neighbouring molecule (*Cg* is the centroid of the C26–C31 isopropoxyphenyl ring).

### Experimental

The title compound was purchased from the Dr. Ehrenstorfer GmbH Company. Single crystals suitable for X-ray diffraction were prepared by slow evaporation of a solution of the title compound in dichloromethane at room temperature.

### Refinement

All H atoms were positioned geometrically and refined using a riding model with d(C—H) = 0.88 Å, *U*_iso_ = 1.2*U*_eq_(C) for amide, d(C—H) = 0.95 Å, *U*_iso_ = 1.2*U*_eq_(C) for aromatic, d(C—H) = 1.00 Å, *U*_iso_ = 1.2*U*_eq_(C) for tertiary, and d(C—H) = 0.98 Å, *U*_iso_ = 1.5*U*_eq_(C) for methyl protons.

### Crystal data

**Table:**

C_17_H_16_F_3_NO_2_	*Z* = 4
*M**_r_* = 323.31	*F*(000) = 672
Triclinic, *P*1	*D*_x_ = 1.363 Mg m^−^^3^
Hall symbol: -P 1	Mo *K*α radiation, λ = 0.71073 Å
*a* = 9.3264 (3) Å	Cell parameters from 5677 reflections
*b* = 11.9326 (3) Å	θ = 2.2–28.3°
*c* = 15.2208 (3) Å	µ = 0.11 mm^−^^1^
α = 71.125 (2)°	*T* = 173 K
β = 87.493 (2)°	Block, colourless
γ = 79.495 (2)°	0.29 × 0.25 × 0.20 mm
*V* = 1575.73 (7) Å^3^	

### Data collection

**Table:**

Bruker SMART APEXII CCD diffractometer	6778 independent reflections
Radiation source: rotating anode	5306 reflections with *I* > 2σ(*I*)
graphite multilayer	*R*_int_ = 0.025
Detector resolution: 10.0 pixels mm^-1^	θ_max_ = 27.0°, θ_min_ = 1.4°
φ and ω scans	*h* = −9→11
Absorption correction: multi-scan (*SADABS*; Sheldrick, 1996)	*k* = −15→15
*T*_min_ = 0.968, *T*_max_ = 0.978	*l* = −19→19
14333 measured reflections	

### Refinement

**Table:**

Refinement on *F*^2^	Primary atom site location: structure-invariant direct methods
Least-squares matrix: full	Secondary atom site location: difference Fourier map
*R*[*F*^2^ > 2σ(*F*^2^)] = 0.042	Hydrogen site location: difference Fourier map
*wR*(*F*^2^) = 0.111	H-atom parameters constrained
*S* = 1.08	*w* = 1/[σ^2^(*F*_o_^2^) + (0.050*P*)^2^ + 0.242*P*] where *P* = (*F*_o_^2^ + 2*F*_c_^2^)/3
6778 reflections	(Δ/σ)_max_ < 0.001
419 parameters	Δρ_max_ = 0.21 e Å^−^^3^
0 restraints	Δρ_min_ = −0.25 e Å^−^^3^

### Special details

**Table:**

Geometry. All e.s.d.'s (except the e.s.d. in the dihedral angle between two l.s. planes) are estimated using the full covariance matrix. The cell e.s.d.'s are taken into account individually in the estimation of e.s.d.'s in distances, angles and torsion angles; correlations between e.s.d.'s in cell parameters are only used when they are defined by crystal symmetry. An approximate (isotropic) treatment of cell e.s.d.'s is used for estimating e.s.d.'s involving l.s. planes.
Refinement. Refinement of *F*^2^ against ALL reflections. The weighted *R*-factor *wR* and goodness of fit *S* are based on *F*^2^, conventional *R*-factors *R* are based on *F*, with *F* set to zero for negative *F*^2^. The threshold expression of *F*^2^ > σ(*F*^2^) is used only for calculating *R*-factors(gt) *etc*. and is not relevant to the choice of reflections for refinement. *R*-factors based on *F*^2^ are statistically about twice as large as those based on *F*, and *R*- factors based on ALL data will be even larger.

### Fractional atomic coordinates and isotropic or equivalent isotropic displacement parameters (Å^2^)

**Table:**

	*x*	*y*	*z*	*U*_iso_*/*U*_eq_	
F1	0.72396 (12)	−0.13855 (11)	0.10482 (7)	0.0653 (3)	
F2	0.93326 (12)	−0.10004 (10)	0.06050 (6)	0.0579 (3)	
F3	0.88346 (16)	−0.27044 (10)	0.06979 (7)	0.0742 (4)	
F4	0.41964 (12)	−0.27986 (8)	0.40792 (6)	0.0533 (3)	
F5	0.63192 (12)	−0.34713 (11)	0.36852 (7)	0.0676 (3)	
F6	0.46092 (15)	−0.44715 (9)	0.37974 (8)	0.0694 (3)	
O1	1.05303 (11)	0.01411 (10)	0.20277 (8)	0.0416 (3)	
O2	0.44736 (11)	0.40242 (8)	0.14189 (7)	0.0356 (2)	
O3	0.56201 (10)	−0.06249 (9)	0.29773 (7)	0.0358 (2)	
O4	−0.01982 (11)	0.27137 (9)	0.40762 (7)	0.0365 (2)	
N1	0.80616 (12)	0.03637 (10)	0.19997 (8)	0.0311 (3)	
H1N	0.7330	−0.0033	0.2141	0.037*	
N2	0.31422 (12)	−0.00838 (10)	0.29585 (7)	0.0278 (2)	
H2N	0.2346	−0.0189	0.2729	0.033*	
C1	0.86575 (18)	−0.18719 (15)	0.11217 (10)	0.0411 (4)	
C2	0.92024 (15)	−0.23796 (13)	0.21080 (9)	0.0330 (3)	
C3	0.94306 (18)	−0.36147 (14)	0.25362 (11)	0.0416 (4)	
H3	0.9202	−0.4119	0.2211	0.050*	
C4	0.9986 (2)	−0.41184 (15)	0.34305 (11)	0.0488 (4)	
H4	1.0137	−0.4966	0.3719	0.059*	
C5	1.03223 (19)	−0.33923 (15)	0.39034 (11)	0.0482 (4)	
H5	1.0710	−0.3738	0.4518	0.058*	
C6	1.00960 (17)	−0.21587 (15)	0.34832 (10)	0.0406 (4)	
H6	1.0336	−0.1662	0.3813	0.049*	
C7	0.95242 (14)	−0.16344 (13)	0.25875 (9)	0.0304 (3)	
C8	0.94110 (15)	−0.02947 (13)	0.21688 (9)	0.0307 (3)	
C9	0.76977 (15)	0.16387 (12)	0.16153 (9)	0.0287 (3)	
C10	0.86874 (16)	0.23390 (14)	0.11281 (10)	0.0375 (3)	
H10	0.9651	0.1980	0.1030	0.045*	
C11	0.82379 (18)	0.35794 (15)	0.07864 (10)	0.0426 (4)	
H11	0.8917	0.4069	0.0468	0.051*	
C12	0.68377 (17)	0.41177 (13)	0.08955 (10)	0.0371 (3)	
H12	0.6556	0.4968	0.0655	0.045*	
C13	0.58392 (15)	0.34064 (12)	0.13603 (9)	0.0295 (3)	
C14	0.62674 (15)	0.21664 (12)	0.17305 (9)	0.0282 (3)	
H14	0.5591	0.1681	0.2060	0.034*	
C15	0.32963 (16)	0.33676 (12)	0.17713 (10)	0.0344 (3)	
H15	0.3616	0.2710	0.2365	0.041*	
C16	0.20828 (19)	0.42797 (15)	0.19619 (12)	0.0506 (5)	
H16A	0.2392	0.4548	0.2456	0.076*	
H16B	0.1215	0.3912	0.2157	0.076*	
H16C	0.1853	0.4972	0.1396	0.076*	
C17	0.28423 (18)	0.28361 (15)	0.10776 (11)	0.0438 (4)	
H17A	0.2418	0.3486	0.0525	0.066*	
H17B	0.2117	0.2329	0.1355	0.066*	
H17C	0.3697	0.2347	0.0901	0.066*	
C18	0.49043 (19)	−0.33559 (14)	0.35097 (11)	0.0433 (4)	
C19	0.44895 (16)	−0.27094 (13)	0.25121 (10)	0.0355 (3)	
C20	0.4374 (2)	−0.33761 (15)	0.19284 (12)	0.0485 (4)	
H20	0.4584	−0.4230	0.2162	0.058*	
C21	0.3958 (2)	−0.28018 (18)	0.10115 (13)	0.0565 (5)	
H21	0.3891	−0.3262	0.0614	0.068*	
C22	0.3637 (2)	−0.15650 (18)	0.06703 (12)	0.0539 (5)	
H22	0.3335	−0.1173	0.0040	0.065*	
C23	0.37535 (18)	−0.08950 (15)	0.12429 (10)	0.0412 (4)	
H23	0.3528	−0.0042	0.1004	0.049*	
C24	0.41967 (14)	−0.14543 (12)	0.21644 (9)	0.0297 (3)	
C25	0.44013 (15)	−0.06876 (12)	0.27467 (9)	0.0275 (3)	
C26	0.29226 (14)	0.06987 (11)	0.35002 (8)	0.0254 (3)	
C27	0.14916 (15)	0.12932 (11)	0.35161 (9)	0.0268 (3)	
H27	0.0743	0.1161	0.3177	0.032*	
C28	0.11661 (15)	0.20774 (12)	0.40283 (9)	0.0290 (3)	
C29	0.22679 (16)	0.22750 (13)	0.45182 (9)	0.0348 (3)	
H29	0.2054	0.2817	0.4865	0.042*	
C30	0.36701 (17)	0.16775 (13)	0.44951 (10)	0.0351 (3)	
H30	0.4417	0.1813	0.4833	0.042*	
C31	0.40278 (15)	0.08807 (12)	0.39927 (9)	0.0297 (3)	
H31	0.5001	0.0472	0.3987	0.036*	
C32	−0.14292 (15)	0.24713 (13)	0.36633 (9)	0.0323 (3)	
H32	−0.1172	0.2439	0.3026	0.039*	
C33	−0.18436 (19)	0.12950 (15)	0.42444 (12)	0.0493 (4)	
H33A	−0.1006	0.0643	0.4307	0.074*	
H33B	−0.2658	0.1138	0.3943	0.074*	
H33C	−0.2136	0.1338	0.4861	0.074*	
C34	−0.26441 (18)	0.35293 (15)	0.35933 (11)	0.0451 (4)	
H34A	−0.2836	0.3607	0.4211	0.068*	
H34B	−0.3529	0.3398	0.3346	0.068*	
H34C	−0.2354	0.4267	0.3178	0.068*	

### Atomic displacement parameters (Å^2^)

**Table:**

	*U*^11^	*U*^22^	*U*^33^	*U*^12^	*U*^13^	*U*^23^
F1	0.0450 (6)	0.0899 (9)	0.0554 (6)	−0.0044 (6)	−0.0153 (5)	−0.0178 (6)
F2	0.0718 (7)	0.0689 (7)	0.0350 (5)	−0.0267 (6)	0.0019 (5)	−0.0119 (5)
F3	0.1204 (11)	0.0642 (7)	0.0508 (6)	−0.0126 (7)	−0.0116 (6)	−0.0364 (5)
F4	0.0734 (7)	0.0427 (5)	0.0395 (5)	−0.0005 (5)	0.0080 (5)	−0.0137 (4)
F5	0.0504 (7)	0.0775 (8)	0.0618 (6)	0.0063 (6)	−0.0135 (5)	−0.0123 (6)
F6	0.1035 (10)	0.0319 (5)	0.0681 (7)	−0.0118 (6)	0.0020 (6)	−0.0100 (5)
O1	0.0247 (6)	0.0436 (6)	0.0585 (7)	−0.0057 (5)	−0.0061 (5)	−0.0187 (5)
O2	0.0344 (6)	0.0246 (5)	0.0453 (5)	−0.0040 (4)	0.0047 (4)	−0.0089 (4)
O3	0.0222 (5)	0.0448 (6)	0.0462 (6)	−0.0057 (4)	0.0003 (4)	−0.0226 (5)
O4	0.0299 (5)	0.0406 (6)	0.0459 (5)	−0.0004 (4)	−0.0003 (4)	−0.0264 (5)
N1	0.0234 (6)	0.0320 (6)	0.0390 (6)	−0.0047 (5)	0.0000 (5)	−0.0132 (5)
N2	0.0220 (6)	0.0293 (6)	0.0359 (6)	−0.0040 (4)	−0.0029 (4)	−0.0156 (5)
C1	0.0435 (9)	0.0461 (9)	0.0404 (8)	−0.0114 (7)	−0.0014 (7)	−0.0209 (7)
C2	0.0283 (7)	0.0371 (8)	0.0352 (7)	−0.0016 (6)	0.0031 (6)	−0.0165 (6)
C3	0.0451 (9)	0.0372 (8)	0.0468 (8)	−0.0040 (7)	0.0063 (7)	−0.0218 (7)
C4	0.0545 (10)	0.0364 (8)	0.0466 (9)	0.0048 (7)	0.0085 (8)	−0.0093 (7)
C5	0.0502 (10)	0.0506 (10)	0.0345 (7)	0.0067 (8)	−0.0030 (7)	−0.0091 (7)
C6	0.0403 (9)	0.0470 (9)	0.0357 (7)	−0.0006 (7)	−0.0041 (6)	−0.0181 (7)
C7	0.0222 (7)	0.0362 (7)	0.0338 (7)	−0.0003 (5)	0.0002 (5)	−0.0154 (6)
C8	0.0250 (7)	0.0390 (8)	0.0331 (7)	−0.0035 (6)	−0.0028 (5)	−0.0189 (6)
C9	0.0289 (7)	0.0317 (7)	0.0279 (6)	−0.0056 (5)	−0.0033 (5)	−0.0123 (5)
C10	0.0282 (8)	0.0451 (9)	0.0364 (7)	−0.0067 (6)	0.0024 (6)	−0.0094 (6)
C11	0.0388 (9)	0.0442 (9)	0.0406 (8)	−0.0166 (7)	0.0041 (7)	−0.0034 (7)
C12	0.0412 (9)	0.0311 (7)	0.0363 (7)	−0.0101 (6)	−0.0012 (6)	−0.0051 (6)
C13	0.0324 (8)	0.0306 (7)	0.0268 (6)	−0.0061 (6)	−0.0011 (5)	−0.0102 (5)
C14	0.0276 (7)	0.0291 (7)	0.0301 (6)	−0.0080 (5)	0.0002 (5)	−0.0107 (5)
C15	0.0323 (8)	0.0271 (7)	0.0374 (7)	−0.0029 (6)	0.0047 (6)	−0.0035 (6)
C16	0.0435 (10)	0.0376 (8)	0.0577 (10)	0.0044 (7)	0.0133 (8)	−0.0057 (7)
C17	0.0381 (9)	0.0412 (9)	0.0490 (9)	−0.0094 (7)	−0.0027 (7)	−0.0088 (7)
C18	0.0468 (10)	0.0314 (8)	0.0499 (9)	−0.0016 (7)	0.0040 (7)	−0.0138 (7)
C19	0.0301 (8)	0.0350 (8)	0.0452 (8)	−0.0027 (6)	0.0025 (6)	−0.0200 (6)
C20	0.0529 (10)	0.0396 (9)	0.0614 (10)	−0.0045 (7)	0.0019 (8)	−0.0295 (8)
C21	0.0642 (12)	0.0623 (12)	0.0597 (11)	−0.0069 (9)	−0.0044 (9)	−0.0439 (9)
C22	0.0592 (12)	0.0651 (12)	0.0423 (9)	0.0003 (9)	−0.0109 (8)	−0.0284 (8)
C23	0.0428 (9)	0.0405 (8)	0.0405 (8)	0.0017 (7)	−0.0056 (7)	−0.0172 (7)
C24	0.0212 (7)	0.0341 (7)	0.0371 (7)	−0.0024 (5)	0.0008 (5)	−0.0174 (6)
C25	0.0246 (7)	0.0269 (7)	0.0304 (6)	−0.0035 (5)	0.0008 (5)	−0.0090 (5)
C26	0.0284 (7)	0.0225 (6)	0.0243 (6)	−0.0048 (5)	0.0002 (5)	−0.0060 (5)
C27	0.0264 (7)	0.0259 (6)	0.0291 (6)	−0.0053 (5)	−0.0008 (5)	−0.0098 (5)
C28	0.0297 (7)	0.0282 (7)	0.0293 (6)	−0.0038 (5)	0.0011 (5)	−0.0103 (5)
C29	0.0403 (8)	0.0355 (8)	0.0336 (7)	−0.0043 (6)	−0.0015 (6)	−0.0190 (6)
C30	0.0348 (8)	0.0395 (8)	0.0345 (7)	−0.0075 (6)	−0.0075 (6)	−0.0155 (6)
C31	0.0274 (7)	0.0313 (7)	0.0300 (6)	−0.0042 (5)	−0.0038 (5)	−0.0093 (5)
C32	0.0296 (7)	0.0351 (7)	0.0329 (7)	−0.0017 (6)	−0.0004 (6)	−0.0141 (6)
C33	0.0372 (9)	0.0466 (9)	0.0577 (10)	−0.0097 (7)	0.0039 (7)	−0.0070 (8)
C34	0.0373 (9)	0.0466 (9)	0.0495 (9)	0.0064 (7)	−0.0015 (7)	−0.0202 (7)

### Geometric parameters (Å, °)

**Table:**

F1—C1	1.3375 (19)	C15—C17	1.506 (2)
F2—C1	1.3276 (19)	C15—C16	1.5087 (19)
F3—C1	1.3325 (18)	C15—H15	1.0000
F4—C18	1.3387 (17)	C16—H16A	0.9800
F5—C18	1.331 (2)	C16—H16B	0.9800
F6—C18	1.3362 (19)	C16—H16C	0.9800
O1—C8	1.2298 (18)	C17—H17A	0.9800
O2—C13	1.3652 (16)	C17—H17B	0.9800
O2—C15	1.4482 (17)	C17—H17C	0.9800
O3—C25	1.2264 (17)	C18—C19	1.495 (2)
O4—C28	1.3697 (16)	C19—C20	1.389 (2)
O4—C32	1.4486 (18)	C19—C24	1.396 (2)
N1—C8	1.3433 (17)	C20—C21	1.378 (3)
N1—C9	1.4231 (17)	C20—H20	0.9500
N1—H1N	0.8800	C21—C22	1.376 (3)
N2—C25	1.3456 (16)	C21—H21	0.9500
N2—C26	1.4167 (16)	C22—C23	1.379 (2)
N2—H2N	0.8800	C22—H22	0.9500
C1—C2	1.498 (2)	C23—C24	1.388 (2)
C2—C3	1.385 (2)	C23—H23	0.9500
C2—C7	1.396 (2)	C24—C25	1.5041 (19)
C3—C4	1.379 (2)	C26—C31	1.388 (2)
C3—H3	0.9500	C26—C27	1.3957 (18)
C4—C5	1.374 (3)	C27—C28	1.3869 (18)
C4—H4	0.9500	C27—H27	0.9500
C5—C6	1.382 (2)	C28—C29	1.392 (2)
C5—H5	0.9500	C29—C30	1.376 (2)
C6—C7	1.387 (2)	C29—H29	0.9500
C6—H6	0.9500	C30—C31	1.3902 (19)
C7—C8	1.503 (2)	C30—H30	0.9500
C9—C10	1.386 (2)	C31—H31	0.9500
C9—C14	1.3969 (18)	C32—C33	1.506 (2)
C10—C11	1.390 (2)	C32—C34	1.5136 (19)
C10—H10	0.9500	C32—H32	1.0000
C11—C12	1.375 (2)	C33—H33A	0.9800
C11—H11	0.9500	C33—H33B	0.9800
C12—C13	1.389 (2)	C33—H33C	0.9800
C12—H12	0.9500	C34—H34A	0.9800
C13—C14	1.3896 (18)	C34—H34B	0.9800
C14—H14	0.9500	C34—H34C	0.9800
			
C13—O2—C15	119.52 (10)	H17A—C17—H17B	109.5
C28—O4—C32	119.39 (10)	C15—C17—H17C	109.5
C8—N1—C9	126.46 (12)	H17A—C17—H17C	109.5
C8—N1—H1N	116.8	H17B—C17—H17C	109.5
C9—N1—H1N	116.8	F5—C18—F6	105.95 (13)
C25—N2—C26	128.88 (12)	F5—C18—F4	106.07 (14)
C25—N2—H2N	115.6	F6—C18—F4	106.21 (13)
C26—N2—H2N	115.6	F5—C18—C19	113.22 (13)
F2—C1—F3	106.16 (12)	F6—C18—C19	112.24 (14)
F2—C1—F1	105.28 (13)	F4—C18—C19	112.59 (12)
F3—C1—F1	106.78 (14)	C20—C19—C24	119.76 (14)
F2—C1—C2	113.10 (14)	C20—C19—C18	119.06 (14)
F3—C1—C2	112.14 (13)	C24—C19—C18	121.17 (13)
F1—C1—C2	112.82 (12)	C21—C20—C19	120.20 (16)
C3—C2—C7	119.83 (13)	C21—C20—H20	119.9
C3—C2—C1	118.94 (14)	C19—C20—H20	119.9
C7—C2—C1	121.19 (13)	C22—C21—C20	120.27 (16)
C4—C3—C2	120.60 (15)	C22—C21—H21	119.9
C4—C3—H3	119.7	C20—C21—H21	119.9
C2—C3—H3	119.7	C21—C22—C23	119.98 (16)
C5—C4—C3	119.92 (15)	C21—C22—H22	120.0
C5—C4—H4	120.0	C23—C22—H22	120.0
C3—C4—H4	120.0	C22—C23—C24	120.71 (15)
C4—C5—C6	119.93 (15)	C22—C23—H23	119.6
C4—C5—H5	120.0	C24—C23—H23	119.6
C6—C5—H5	120.0	C23—C24—C19	119.06 (13)
C5—C6—C7	120.99 (15)	C23—C24—C25	118.88 (13)
C5—C6—H6	119.5	C19—C24—C25	122.02 (12)
C7—C6—H6	119.5	O3—C25—N2	125.01 (13)
C6—C7—C2	118.71 (14)	O3—C25—C24	121.40 (11)
C6—C7—C8	116.96 (13)	N2—C25—C24	113.57 (12)
C2—C7—C8	124.13 (12)	C31—C26—C27	120.81 (12)
O1—C8—N1	123.74 (13)	C31—C26—N2	123.73 (12)
O1—C8—C7	119.35 (12)	C27—C26—N2	115.47 (12)
N1—C8—C7	116.87 (12)	C28—C27—C26	119.78 (13)
C10—C9—C14	120.61 (13)	C28—C27—H27	120.1
C10—C9—N1	122.53 (12)	C26—C27—H27	120.1
C14—C9—N1	116.85 (12)	O4—C28—C27	124.05 (13)
C9—C10—C11	118.45 (13)	O4—C28—C29	116.01 (12)
C9—C10—H10	120.8	C27—C28—C29	119.92 (12)
C11—C10—H10	120.8	C30—C29—C28	119.36 (13)
C12—C11—C10	121.86 (14)	C30—C29—H29	120.3
C12—C11—H11	119.1	C28—C29—H29	120.3
C10—C11—H11	119.1	C29—C30—C31	122.02 (14)
C11—C12—C13	119.33 (14)	C29—C30—H30	119.0
C11—C12—H12	120.3	C31—C30—H30	119.0
C13—C12—H12	120.3	C26—C31—C30	118.11 (13)
O2—C13—C12	114.83 (12)	C26—C31—H31	120.9
O2—C13—C14	125.07 (12)	C30—C31—H31	120.9
C12—C13—C14	120.10 (13)	O4—C32—C33	110.87 (12)
C13—C14—C9	119.60 (12)	O4—C32—C34	105.56 (12)
C13—C14—H14	120.2	C33—C32—C34	112.17 (13)
C9—C14—H14	120.2	O4—C32—H32	109.4
O2—C15—C17	111.00 (11)	C33—C32—H32	109.4
O2—C15—C16	104.97 (12)	C34—C32—H32	109.4
C17—C15—C16	112.11 (14)	C32—C33—H33A	109.5
O2—C15—H15	109.6	C32—C33—H33B	109.5
C17—C15—H15	109.6	H33A—C33—H33B	109.5
C16—C15—H15	109.6	C32—C33—H33C	109.5
C15—C16—H16A	109.5	H33A—C33—H33C	109.5
C15—C16—H16B	109.5	H33B—C33—H33C	109.5
H16A—C16—H16B	109.5	C32—C34—H34A	109.5
C15—C16—H16C	109.5	C32—C34—H34B	109.5
H16A—C16—H16C	109.5	H34A—C34—H34B	109.5
H16B—C16—H16C	109.5	C32—C34—H34C	109.5
C15—C17—H17A	109.5	H34A—C34—H34C	109.5
C15—C17—H17B	109.5	H34B—C34—H34C	109.5
			
F2—C1—C2—C3	−137.20 (14)	F5—C18—C19—C20	97.18 (18)
F3—C1—C2—C3	−17.2 (2)	F6—C18—C19—C20	−22.7 (2)
F1—C1—C2—C3	103.43 (16)	F4—C18—C19—C20	−142.51 (15)
F2—C1—C2—C7	40.77 (19)	F5—C18—C19—C24	−83.80 (17)
F3—C1—C2—C7	160.79 (13)	F6—C18—C19—C24	156.30 (14)
F1—C1—C2—C7	−78.60 (18)	F4—C18—C19—C24	36.5 (2)
C7—C2—C3—C4	−0.6 (2)	C24—C19—C20—C21	−0.7 (3)
C1—C2—C3—C4	177.40 (14)	C18—C19—C20—C21	178.34 (16)
C2—C3—C4—C5	−0.2 (2)	C19—C20—C21—C22	−0.7 (3)
C3—C4—C5—C6	0.3 (2)	C20—C21—C22—C23	1.0 (3)
C4—C5—C6—C7	0.4 (2)	C21—C22—C23—C24	0.2 (3)
C5—C6—C7—C2	−1.1 (2)	C22—C23—C24—C19	−1.6 (2)
C5—C6—C7—C8	−176.10 (13)	C22—C23—C24—C25	176.12 (15)
C3—C2—C7—C6	1.2 (2)	C20—C19—C24—C23	1.8 (2)
C1—C2—C7—C6	−176.73 (13)	C18—C19—C24—C23	−177.19 (14)
C3—C2—C7—C8	175.82 (13)	C20—C19—C24—C25	−175.80 (14)
C1—C2—C7—C8	−2.1 (2)	C18—C19—C24—C25	5.2 (2)
C9—N1—C8—O1	1.9 (2)	C26—N2—C25—O3	−2.4 (2)
C9—N1—C8—C7	179.49 (11)	C26—N2—C25—C24	178.95 (12)
C6—C7—C8—O1	64.09 (17)	C23—C24—C25—O3	−107.63 (16)
C2—C7—C8—O1	−110.60 (16)	C19—C24—C25—O3	70.00 (18)
C6—C7—C8—N1	−113.65 (15)	C23—C24—C25—N2	71.07 (16)
C2—C7—C8—N1	71.67 (17)	C19—C24—C25—N2	−111.31 (14)
C8—N1—C9—C10	18.6 (2)	C25—N2—C26—C31	−6.7 (2)
C8—N1—C9—C14	−162.87 (13)	C25—N2—C26—C27	173.54 (12)
C14—C9—C10—C11	2.2 (2)	C31—C26—C27—C28	0.10 (18)
N1—C9—C10—C11	−179.32 (13)	N2—C26—C27—C28	179.91 (11)
C9—C10—C11—C12	−1.9 (2)	C32—O4—C28—C27	7.48 (19)
C10—C11—C12—C13	0.0 (2)	C32—O4—C28—C29	−173.89 (12)
C15—O2—C13—C12	171.40 (12)	C26—C27—C28—O4	179.05 (11)
C15—O2—C13—C14	−9.2 (2)	C26—C27—C28—C29	0.47 (19)
C11—C12—C13—O2	−178.99 (13)	O4—C28—C29—C30	−179.33 (12)
C11—C12—C13—C14	1.6 (2)	C27—C28—C29—C30	−0.6 (2)
O2—C13—C14—C9	179.35 (12)	C28—C29—C30—C31	0.2 (2)
C12—C13—C14—C9	−1.3 (2)	C27—C26—C31—C30	−0.48 (19)
C10—C9—C14—C13	−0.6 (2)	N2—C26—C31—C30	179.73 (12)
N1—C9—C14—C13	−179.16 (12)	C29—C30—C31—C26	0.3 (2)
C13—O2—C15—C17	−72.29 (16)	C28—O4—C32—C33	73.95 (15)
C13—O2—C15—C16	166.38 (12)	C28—O4—C32—C34	−164.36 (12)

### Hydrogen-bond geometry (Å, °)

**Table:**

Cg is the centroid of the C26–C31 isopropoxyphenyl ring.

**Table:**

*D*—H···*A*	*D*—H	H···*A*	*D*···*A*	*D*—H···*A*
N1—H1N···O3	0.88	2.08	2.8875 (15)	153
N2—H2N···O1^i^	0.88	1.95	2.7984 (16)	161
C15—H15···Cg	1.00	2.69	3.53	142

Symmetry codes: (i) *x*−1, *y*, *z*.
